# Genomic and Metabolomic Analyses of the Marine Fungus *Emericellopsis cladophorae*: Insights into Saltwater Adaptability Mechanisms and Its Biosynthetic Potential

**DOI:** 10.3390/jof8010031

**Published:** 2021-12-30

**Authors:** Micael F. M. Gonçalves, Sandra Hilário, Yves Van de Peer, Ana C. Esteves, Artur Alves

**Affiliations:** 1 CESAM, Department of Biology, University of Aveiro, 3810-193 Aveiro, Portugal; mfmg@ua.pt (M.F.M.G.); sandra.hilario@ua.pt (S.H.); artur.alves@ua.pt (A.A.); 2 Department of Plant Biotechnology and Bioinformatics, Ghent University, 9052 Ghent, Belgium; yves.vandepeer@psb.vib-ugent.be; 3 Center for Plant Systems Biology, VIB, 9052 Ghent, Belgium; 4 Department of Biochemistry, Genetics and Microbiology, University of Pretoria, Pretoria 0028, South Africa; 5 College of Horticulture, Academy for Advanced Interdisciplinary Studies, Nanjing Agricultural University, Nanjing 210095, China

**Keywords:** antimicrobial, anticancer, marine fungi, metabolites, whole genome sequencing

## Abstract

The genus *Emericellopsis* is found in terrestrial, but mainly in marine, environments with a worldwide distribution. Although *Emericellopsis* has been recognized as an important source of bioactive compounds, the range of metabolites expressed by the species of this genus, as well as the genes involved in their production are still poorly known. Untargeted metabolomics, using UPLC- QToF–MS/MS, and genome sequencing (Illumina HiSeq) was performed to unlock *E*. *cladophorae* MUM 19.33 chemical diversity. The genome of *E*. *cladophorae* is 26.9 Mb and encodes 8572 genes. A large set of genes encoding carbohydrate-active enzymes (CAZymes), secreted proteins, transporters, and secondary metabolite biosynthetic gene clusters were identified. Our analysis also revealed genomic signatures that may reflect a certain fungal adaptability to the marine environment, such as genes encoding for (1) the high-osmolarity glycerol pathway; (2) osmolytes’ biosynthetic processes; (3) ion transport systems, and (4) CAZymes classes allowing the utilization of marine polysaccharides. The fungal crude extract library constructed revealed a promising source of antifungal (e.g., 9,12,13-Trihydroxyoctadec-10-enoic acid, hymeglusin), antibacterial (e.g., NovobiocinA), anticancer (e.g., daunomycinone, isoreserpin, flavopiridol), and anti-inflammatory (e.g., 2’-O-Galloylhyperin) metabolites. We also detected unknown compounds with no structural match in the databases used. The metabolites’ profiles of *E*. *cladophorae* MUM 19.33 fermentations were salt dependent. The results of this study contribute to unravel aspects of the biology and ecology of this marine fungus. The genome and metabolome data are relevant for future biotechnological exploitation of the species.

## 1. Introduction

The genus *Emericellopsis* was introduced by Kingma [1] and is distributed worldwide. Species of *Emericellopsis* can be found in association with seaweeds in estuarine or marine habitats, but also in soils, peat, and rhizomes [2,3,4]. However, it is in saline environments that *Emericellopsis* species appear more frequently and thrive: macroalgae, sponges, sea and estuarine waters, marine sediments and even in soils with periodic flooding and extreme humidity and alkalinity [2,3,4,5,6,7].

It has been demonstrated that species of *Emericellopsis* are important sources of bioactive metabolites, such as peptaibols with antibacterial and antifungal activities [8,9]. The Norine database [10], which is dedicated to non-ribosomal peptide synthases (NRPS), includes some of these compounds: the antiamoebins I–XI from *E*. *salmosynnemata* and *E*. *synnematicola*, the bergofungins A–D from *E*. *donezkii*, the emerimicins II–IV from *E*. *microspora* and *E*. *minima*, the heptaibin from *Emericellopsis* sp. BAUA8289 and the zervamicins from *E*. *salmosynnemata* [11,12,13,14,15,16,17]. Recently, Kuvarina et al. [8] reported emericellipsins A–E as novel peptaibols from *E*. *alkalina*.

*Emericellopsis cladophorae* was isolated from the reticulated filamentous green alga *Cladophora* sp. at the estuary Ria de Aveiro, Portugal, during the summer of 2018 [7]. Crude extracts from *E*. *cladophorae* have antibacterial, antioxidant and cytotoxic properties [18]. Moreover, it was shown that *E*. *cladophorae* strain MUM 19.33 produces proteinases, cellulases, chitinases, pectinases, pectin lyases and ureases, among other enzymatic activities. Also, it was shown that all the bioactivity profiles assayed are salt-dependent, i.e., the presence or absence of sea salt in culture media induces alteration in the metabolome and consequently in the biological activities of *E*. *cladophorae*.

Fungal genome sequencing and metabolomics analyses have become more common and facilitated the research of gene diversity, helping to understand gene functions, pathogenicity, and the identification of secondary metabolites [19]. However, crucial information about marine fungi genomes’ structure remains poorly explored. One reason is the shortage of fungal reference genomes derived from the marine environment in public databases, essential for gene annotation algorithms. In addition, there are only few studies on the metabolome of marine fungi [20,21]. At present, studies on *Emericellopsis* mostly focus on the phylogenetic identification of species, but information about the entire genome and metabolome of this fungal genus is lacking. Recently, Hagestad et al. [22] provided a detailed taxonomic and genomic description of the first sequenced *Emericellopsis* species: *E*. *atlantica*, isolated from the sponge *Stelletta normani* in the Atlantic Ocean.

The aim of this study was mining the genome and metabolome of *E*. *cladophorae* strain MUM 19.33 to disclose its biosynthetic potential, and, as well, for carbohydrate-active enzymes, transporters, and secreted proteins, among several others. The data generated in this study contribute to the knowledge of full biotechnological potential and biology of *Emericellopsis* and other marine fungal species.

## 2. Materials and Methods

### 2.1. Culture Conditions and DNA Extraction

Two mycelium-colonized agar plugs were inoculated into Erlenmeyer flasks containing 50 mL of Potato Dextrose Broth (PDB) (Merck, Darmstadt, Germany) at 25 °C, without agitation for seven days, in the dark. Afterwards, mycelium was filtered through sterile filter paper, and was immediately grounded in liquid nitrogen. DNA was extracted according to Pitcher et al. [23]. The quality of the DNA was assessed by agarose gel electrophoresis (0.8%). DNA purity and quantity were determined using a NanoDrop 2000 spectrophotometer (Thermo Fisher Scientific Inc., Waltham, MA, USA).

### 2.2. Genome Sequencing, Assembly, and Prediction

*Emericellopsis cladophorae* strain MUM 19.33 genome was sequenced from 100 ng of genomic DNA by Genome Sequencer Illumina HiSeq (2 × 150 bp paired-end reads) with NovaSeq 6000 S2 PE150 XP platform (Eurofins, Brussels, Belgium). Adapter sequences and low-quality reads were removed from output reads using the Trimmomatic software v.0.39 [24]. The quality assessment analysis of the reads was performed in the fastQC program (Babraham, Bioinformatics, 2016). Then, the nuclear genome was assembled using SPAdes v.3.14 [25]. QUAST web interface (http://cab.cc.spbu.ru/quast/, accessed on 10 January 2021) was used to assess the quality of the assembled genome. Gene prediction of the draft genome assembly was performed using Augustus v.3.3.3 [26] with default parameters and using *Acremonium chrysogenum* gene models as training set.

### 2.3. Genome Annotation and Functional Analysis

Several complementary methodologies were used to annotate the sequences. Dispersed Repeat sequences (DRs) were identified in OmicsBox v.1.4.12 with the Repeat Masking option (RepeatMasker v.4.0.9, accessed on 5 February 2021) [27]. Tandem Repeat sequences (TRs) were identified by Tandem Repeats Finder (TRF) (http://tandem.bu.edu/cgi-bin/trdb/trdb.exe, accessed on 5 February 2021) [28]. Analyses of noncoding RNAs, such as tRNAs were carried out using tRNAscan-SE tool (http://lowelab.ucsc.edu/tRNAscan-SE/, accessed on 5 February 2021) with default parameters [29].

Predicted genes were functionally annotated with OmicsBox using Blast2GO [30] against NCBI’s nonredundant (Nr) database, Gene Ontology (GO), and Kyoto Encyclopedia of Genes and Genomes (KEGG) with an e-value threshold of 1 × 10^−^³. Proteins were classified using InterProScan and the Evolutionary Genealogy of Genes: Non-supervised Orthologous Groups (EggNOG), which also contains the orthologous groups from the original COG/KOG database (euKaryotic cluster of Orthologous Groups of proteins) with an e-value of 1 × 10^−^³.

Carbohydrate-degrading enzymes (CAZymes) were predicted with the web-based application dbCAN (HMMs 5.0) (htttp://www.cazy.org/, accessed on 10 February 2021) using default settings (http://bcb.unl.edu/dbCAN2/blast.php, accessed on 10 February 2021) [31]. Fungal secreted proteins, including signal peptides, were predicted using SignalP [32]. Transporters were identified with a BLAST analysis against the Transporter Classification (TC) Database [33], downloaded in March 2021, with an e-value threshold of 1 × 10^−5^, using the Geneious Prime v.2021.0.3 (htttp://www.geneious.com, accessed on 15 February 2021). The genome has also been screened for the presence of Biosynthetic Gene Clusters (BGC) using the web-based application antiSMASH v.5.0, using strictness ‘relaxed’ option for detection of well-defined and partial clusters containing the functional parts [34].

### 2.4. Comparative Analyses with E. atlantica

The genome of *Emericellopsis cladophorae* MUM 19.33 was compared with the genome of *E*. *atlantica* TS7. To evaluate the genetic and metabolic diversity of both species the information available in JGI Genome Portal database, such as genome size, GC content, CAZymes, BGCs abundance was used.

### 2.5. Small-Scale Fermentation and Extraction of Metabolites

A small-scale fermentation was carried out as described by Gonçalves et al. [18]. Briefly, two plugs of mycelium-colonized agar were inoculated into 1-L Erlenmeyer flasks containing 250 mL of PDB in two conditions: with and without 3% sea salt (Sigma-Aldrich, Darmstadt, Germany) with 4 replicates for each condition. The fungus was grown at 25 °C under stationary conditions for 14 days. Culture media were obtained by filtering the mycelium through sterile filter paper. Then, the culture media was filtrated with 0.45 μm cellulose membrane (GN-6 Metricel, Pall Corporation, New York, NY, USA) followed by 0.2 μm nitrate cellulose membrane (Sartorius Stedim Biotech, Gottingen, Germany) in a vacuum system. Culture media from the 4 replicates were pooled and lyophilized, and dried culture media were weighed and transferred to tubes. Next, 20 mL of cold 80% MeOH (−80 °C) was added to each tube (containing 2 g of lyophilized sample) and vortexed for 5 min. Each mixture was centrifuged at 14,000× *g* for 10 min at 4 °C to remove precipitated proteins. The supernatant was collected, and the extraction process was repeated once. After extraction, the methanolic extracts were filtered using a glass microfiber filter 0.47 mm (Prat Dumas, Couze-St-Front, France), evaporated in vacuo using a rotary evaporator and lyophilized.

For LC-MS, 5 replicates of dried crude extracts (100 mg) for each condition were used. Metabolite extraction was performed by adding MeOH to each of the samples allowing them to vortexed for 40 min. Then, the samples were centrifuged for 5 min at 20,000× *g* and 400 μL of the methanolic fraction was vacuum dried. Afterwards, 100 μL of cyclohexane/water (1/1, *v*/*v*) was added to each sample and vortexed. Each mixture was centrifuged at 20,000× *g* for 5 min and 90 μL of the aqueous phase was filtered on a 96-filter plate and transferred to a 96-well plate. The samples were 10× diluted in water and 10 μL was analyzed by LC-MS.

### 2.6. LC-MS Data Analysis, Processing, and Visualization

UHPLC was performed on an ACQUITY UPLC I-Class system (Waters Corporation, Milford, MA, USA) consisting of a binary pump, a vacuum degasser, an autosampler, and a column oven. Chromatographic separation was carried out on an ACQUITY UPLC BEH C18 column (150 × 2.1 mm, 1.7 μm, Waters Corporation, Milford, MA, USA), and temperature was maintained at 40 °C. A gradient of solution A (99:1:0.1 water:acetonitrile:formic acid, pH 3) and solution B (99:1:0.1 acetonitrile:water:formic acid, pH 3) was used: 99% A for 0.1 min decreased to 50% A in 30 min, decreased to 30% in 5 min, decreased to 0% in 2 min. The flow rate was set to 0.35 mL min^−1^, and the injection volume was 10 μL. The UHPLC system was coupled to a Vion IMS QTOF hybrid mass spectrometer (Waters Corporation, Milford, MA, USA). The LockSpray ion source was operated in negative electrospray ionization mode under the following specific conditions: capillary voltage, 2.5 kV; reference capillary voltage, 3 kV; cone voltage, 40 V; source offset, 50 V; source temperature, 120 °C; desolvation gas temperature, 600 °C; desolvation gas flow, 800 L h^−1^; and cone gas flow, 50 L h^−1^. Mass range was set from 50 to 1000 Da. The collision energy for full HDMSe was set at 6 eV (low energy) and ramped from 20 to 70 eV (high energy), intelligent data capture intensity threshold was set at 5. Nitrogen (greater than 99.5%) was employed as desolvation and cone gas. Leucin-enkephalin (250 pg μL^−1^ in water:acetonitrile 1:1 [*v*/*v*], with 0.1% formic acid) was used for the lock mass calibration, with scanning every 2 min at a scan time of 0.1 s. Profile data were recorded through a UNIFI Scientific Information System (Waters Corporation). Data processing was performed with Progenesis QI software v.2.4 (Waters Corporation). To understand the metabolomic fluctuations in response to sea salt, a IQR (interquartile range) filtering was applied because of a large number of significant ions, resulting in a selection of a set of 2500 ions for the data modeling. The log-transformed and pareto-scaled (normalized) LC-MS integration values of the filtered compound ions were analyzed (data not shown). Principal Component Analysis (PCA), heatmaps, and *t*-test on log-transformed and pareto-scaled (normalized) of the filtered ions were generated and using online MetaboAnalyst v.4.0 software [35]. Computed *p*-values were adjusted using the Benjamin–Hochberg False Discovery Rate (FDR) correction. Ions having an FDR < 0.01 and a log_2_ fold change (FC) >2 or <−2 were considered differently expressed. For identification purposes, the fragmentation data (ESI negative) of the significant ions were selected and matched against in-house library and 44 external spectral libraries (https://mona.fiehnlab.ucdavis.edu/, accessed on 3 December 2020), using MSsearch software. For each ion, the best hit was based on a matching precursor ion (*m*/*z* < 10 ppm difference) and matching fragments (<50 ppm accuracy), generating 5 common fragments, including the precursor *m*/*z*. For each hit, the name of the matching compound followed by the collision energy used, the parent ion as a nominal mass, the chemical formula, a matching factor (MF), a reverse matching factor (RMF), and the name of the library found were obtained (File S1). File S1 contains some positive ionizations, but only ions in negative mode were considered for identification. Thus, annotation was done at level 2 of the Metabolomics Standards Initiative (MSI).

## 3. Results and Discussion

### 3.1. Sequencing, Assembly Data and Genomic Characteristics

General data related to the draft genome of *Emericellopsis cladophorae* MUM 19.33 is presented in Table 1. Briefly, the *E*. *cladophorae* genome size was estimated at 26.7 Mb, assembled in 300 contigs, with 8572 predicted genes from which 41.3% encode for hypothetical proteins, and a GC content of 54.34%.

### 3.2. Repetitive Sequences and of tRNAs

Repetitive sequences are classified as Dispersed Repeats (DRs) and Tandem Repeats (TRs). The total length of the 5258 DRs in *E*. *cladophorae* MUM 19.33 genome is 261,095 bp, covering 0.97% of the genome. With respect to the TRs, there are 2365 sequences with a total length of 232,036 bp covering 0.86% of the genome. 122 tRNAs were also predicted, with a total length of 10,432 bp covering 0.04% of the genome (Table 2). Among the tRNAs, 5 are possible pseudogenes and the remaining 117 anti-codon tRNAs correspond to the 20 common amino acid codons.

### 3.3. Gene Annotation

**Figure 1:**
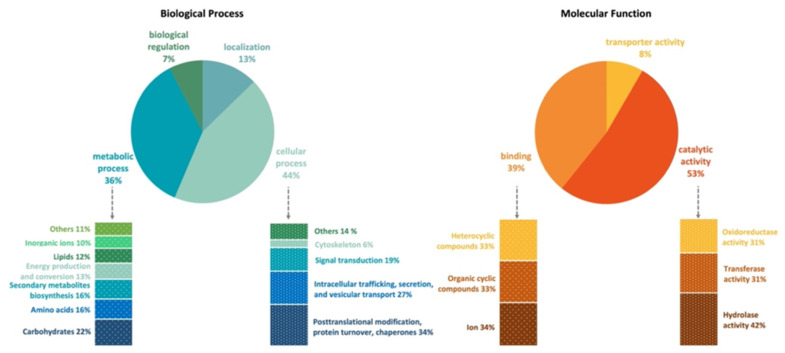
Gene Ontology (GO) functional annotation (pie charts) and EggNOG functional classification (bars charts) of *Emericellopsis cladophorae* MUM 19.33 genome.

The genome of *Emericellopsis cladophorae* MUM 19.33 has 8289 genes annotated according to the NCBI’s nonredundant protein (Nr), UniProt/Swiss-Prot, EggNOG, KEGG, GO, and Pfam databases. There are 7834 (91.4%) cellular proteins and approximately 738 secreted proteins (8.6%) (Tables S1 and S3). Functional analysis (GO, Biological Processes) shows that most genes are involved in cellular (44%) and metabolic process (36%), cellular localization (13%) and biological regulation (7%) and in (GO, Molecular Functions) catalytic (53%), binding (39%), and transporter (8%) activities (Figure 1, Table S1). Genes classified within the “cellular process” category were mainly classified as being involved in posttranslational modification, protein turnover, chaperones (34%); intracellular trafficking, secretion, and vesicular transport (27%); signal transduction (19%); cytoskeleton (6%); and others (14%), which include cell cycle control, cell wall and membrane biogenesis, cell mobility, and defense mechanisms. Within the “metabolic process” category, *E*. *cladophorae* genes are involved in the metabolism and transport of carbohydrates (22%, e.g., starch, sucrose, pyruvate, galactose, fructose, and mannose), amino acids (16%), lipids (12%) and inorganic ions (10%), in the biosynthesis of secondary metabolites (16%, e.g., novobiocin, penicillin, cephalosporin, streptomycin, and carbapenems), and in energy production and conversion (13%). In GO, Molecular Functions, genes are involved in catalytic (53%), binding (39%), and transport (8%) activities (Figure 1, Table S1). These values are in agreement with what has been described in the literature for fungi.

### 3.4. Carbohydrate-Active Enzymes (CAZymes)

There are 407 genes encoding putative CAZymes, from which 203 carry signal peptides were annotated using the HMMER database (Table S2). Among these genes, 200 encode for glycoside hydrolases (GH), 8 for carbohydrate binding modules (CBM), 83 for glycosyltransferases (GT), 72 for auxiliary activities/oxidoreductases (AA), 28 for carbohydrate esterases (CE), and 16 for pectate lyases (PL). The most abundant GH family includes ß-glucosidades (GH3), chitinases (GH18), cellulases (GH5), ß-xylosidades (GH43), amylases (GH13), xyloglucan:xyloglucosyltransferase (GH16), α-glucosidase (GH31) and α-mannosidase (GH47). Regarding GT, UDP-glucuronosyltransferase (GT1), cellulose/chitin synthases (GT2), sucrose synthase (GT4) and fucose-specific ß-1,3-*N*-acetylglucosaminyltransferase (GT31) were the most abundant. Cellobiose dehydrogenase (AA3), glucooligosaccharide/chitooligosaccharide oxidases (AA7) and lipopolysaccharide *N*-acetylglucosaminyltransferase (AA9) which belong to AA family were the most predominant. Within the CBM class, CBM20 involved in starch binding was the most prevalent. In *E*. *cladophorae*, 11 CEs are present with CE5 (acetyl xylan esterase, cutinase) being the most abundant. CE5 participates in the enzymatic hydrolysis of cutin, and are commonly secreted by plant pathogens, enabling them to penetrate through the cuticle [36]. Also, results show that *E*. *cladophorae* genome encodes PL genes such as pectate lyase (PL1), pectate lyase (PL3) and rhamnogalacturonan endolyase (PL4). In marine environment pectin-like polysaccharides have been reported in diatoms, seagrasses, and macro- and microalgae [37,38].

Polysaccharides represent some of the most abundant bioactive substances in marine organisms representing a good resource of nutrients [39]. Thus, the presence of specific CAZymes in *E*. *cladophorae* allows the utilization of polysaccharides—chitin, starch and other marine polysaccharides such as fucoidan and ulvan present in brown and green algae is concordant with *E*. *cladophorae* being a guest of the filamentous green alga *Cladophora* sp. [7].

### 3.5. Transporter Proteins

Transport proteins are classified into five well defined classes according to the transport protein classification (TC) system [33]: channels and pores (TC 1), electrochemical potential-driven transporters (TC 2), primary active transporters (TC 3), group translocators (TC 4), and transmembrane electron carriers (TC 5), accessory factors involved transport (TC 8) and incompletely characterized transport systems (TC 9). *Emericellopsis cladophorae* MUM 19.33 genome encodes transporters (2197 genes) from all the TC classes, accounting for 25.6% of the total predicted genes of *E*. *cladophorae* (Table 3 and Table S4). TC 2 class accounted for 25.9% of transporters in *E*. *cladophorae* genome. This transporters’ class encompass the Major Facilitator Superfamily (MFS), which can transport molecules, controlling membrane homeostasis and regulate internal pH and the stress response machinery in fungi [40]. It has been demonstrated that many of MFS transporters are required for fungi to grow under stress conditions [41] and play an important role in multidrug resistance [42]. Genes encoding transporters of glycerol, inositol, sodium, and chloride were found.

Other transporters’ encoding genes are related to the salt overly sensitive signaling pathway, a well-defined pathway in plants to maintain cellular ion homeostasis by restricting the accumulation of sodium [43]. It is known that at high salinity, fungi maintain osmotic balance mainly by the increased production and accumulation of glycerol or other compatible solutes, such as inositol, mannitol, arabitol, xylitol and nitrogen containing compounds (e.g., glycine, betaine) [44]. *Emericellopsis cladophorae* genome contains genes involved in glycerol, mannitol, inositol, sorbitol, trehalose, glycine, and betaine biosynthetic process, suggesting that *E*. *cladophorae* has adaptability mechanisms to maintain positive turgor pressure allowing the interplay of osmolyte transporters. The second most abundant transporter class in *E*. *cladophorae* is TC 1 accounted for 21% of transporters. These transporters are mainly associated to ionic homeostasis allowing rapid changes in cell physiology [45]. We identify transporters’ encoding genes likely to encode for calcium channels, nucleoporins, aquaporins, among others. Aquaporins are fundamental in all living organisms for maintenance of water equilibrium and their cellular shape and turgor [46]. But to date, little is known about aquaporins in filamentous fungi. In yeasts for example, aquaporins play important roles in establishment of freeze tolerance, spore formation, and cell surface properties for adhesion [47]. We believe that some transporters play a crucial role in ions and osmolytes transport that allow marine fungi to thrive in saltwater, thereby playing roles in nutrient uptake and osmoregulation.

### 3.6. Biosynthetic Gene Clusters

Thirty-seven BGCs involved in the secondary metabolism of *E*. *cladophorae* MUM 19.33 were predicted (Table S5). These gene clusters encode for 7 terpenes, 5 t1PKs and 1 t3PKs (type 1 and 3 polyketide synthases), 10 NRPS, 5 NRPS-t1PKs, 7 NRPS-like, 1 NRPS-like-t1PKs and 1 phosphonate. From the BGCs identified, 3 BGCs have 100% similarity with known BGCs, such as clavaric acid (antitumor), EQ-4 Microperfuranone (immunosuppressive activity) and (-)-Mellein (antifungal). Others BGCs, shared gene similarity with the ascochlorin BGC (87%), cephalosporin C BGC (57% of genes show similarity), leucinostatin A/leucinostatin B BGC (45% of genes show similarity) and with copalyl diphosphate BGC and squalestatin S1 BGC (42% and 40%), respectively. Other genes probably involved in BGC of yanuthone D, oosporein and shearinine D were also detected.

**Figure 2:**
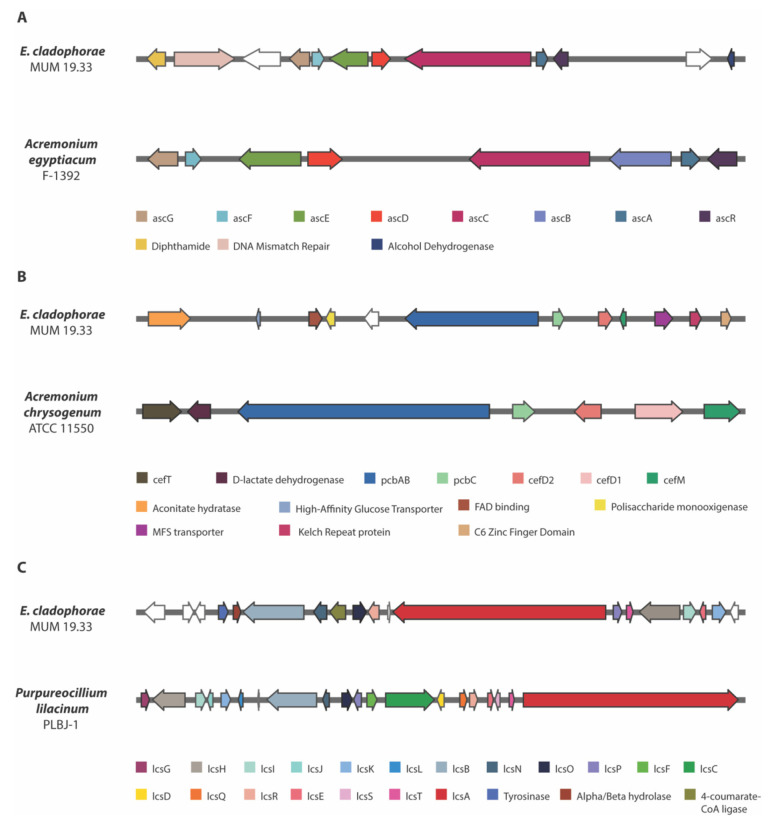
Comparison of three biosynthetic gene regions in *Emericellopsis cladophorae* MUM 19.33 with (**A**) Ascochlorin BGC of *Acremonium egyptiacum* F-1392; (**B**) Cephalosporin C BGC of *Acremonium chrysogenum* ATCC 11,550; and (**C**) Leucinostatin A/B BGC of *Purpureocillium lilacinum* PLBJ-1. The genes that encode hypothetical proteins are represented as white arrows.

Ascochlorin is an isoprenoid antibiotic isolated from *Acremonium egyptiacum* (syn. *A*. *sclerotigenum*), previously known as the phytopathogenic fungus *Ascochyta viciae* [48]. Ascochlorin or related compounds have been reported to show antiviral and antitumor activities [49] and was found in some ascomycetes, including *Acremonium*-like or *Emericellopsis* species [22]. The ascochlorin cluster is constituted by eight genes: *ascA* (prenyltransferase), *ascB* (NRPS-like oxidoreductase), *ascC* (polyketide synthase), *ascD* (halogenase), *ascE* (P450 monooxygenase/P450 reductase), *ascF* (terpene cyclase), *ascG* (cytochrome P450) and *ascR* (transcription regulator). This gene architecture was found in *E*. *cladophorae*, but the *ascB* gene is missing (Figure 2A). Also, near to this cluster other three genes coding for diphthamide, DNA mismatch repair and alcohol dehydrogenase were identified. This similar cluster may indicate that *E*. *cladophorae* can be producer of ascochlorin or a related compound.

Cephalosporins are among the most-widely used drugs for treatment of infections and belongs to the family of beta-lactam antibiotics. From the cephalosporin group, Cephalosporin C is the major source for production of 7-amino cephalosporanic acid (7-ACA) [50]. It has been demonstrated that Cephalosporin C is the major compound produced by *Emericellopsis* species, mainly by *E*. *minima* and *E*. *salmosynnemata* [51]. Cephalosporin C was isolated from *Acremonium chrysogenum* (syn. *Cephalosporium acremonium*) and its biosynthesis is well elucidated [52]. This cluster is comprised by *cefT* (MFS transporter), D-lactate dehydrogenase (ORF 3), *pcbAB* (ACV synthetase), *pcbC* (IPN synthase), *cefD2* (IPN CoA epimerase), *cefD1* (IPN CoA synthetase) and *cefM* (MFS transporter). Also, two other genes, *cefEF* (deacetoxycephalosporin C synthase) and *cefG* (acetyl CoA) were involved in cephalosporin C biosynthesis but belong to a different cluster [53]. In *E*. *cladophorae*, only four genes of Cephalosporin C cluster are present (Figure 2B) namely *pcbAB*, *pcbC*, *cefD1* and *cefM*, which is essential for cephalosporin biosynthesis [53]. Additionally, *cefD2*, *cefT* and D-lactate dehydrogenase lack, but another MFS transporter located near to *cefM* was detected, which may have the same function of *cefT* that is responsible for cephalosporin secretion from the cell.

Leucinostatins are a family of lipopeptide antibiotics isolated firstly from *Purpureocillium lilacinum* [54] with broad extensive biological activities, including antimalarial, antiviral, antibacterial, antifungal, antitumor and phytotoxicity [55]. Compounds such as leucinostatins have also been previously isolated from *Acremonium*-like species [56]. This twenty-genes cluster is constituted by one NRPS (*lcsA*), two PKs (*lcsB* and *lcsC*), phenylacetyl-ligase (*lcsD*), thioesterase (*lcsE*), basic-leucine zipper transcription factor (*lcsF*), sterigmatocystin 8-O-methyltransferase (*lcsG*), two ABC multidrug transporter (*lcsH* and *lcsO*), isotrichodermin C-15 hydroxylase (*lcsI*), thioesterase-like (*lcsJ*), two cytochrome P450 (*lcsK* and *lcsN*), bZIP transcription factor (*lcsL*), hypothetical protein (*lcsM*), branched-chain amino acid aminotransferase (*lcsP*), tRNA synthetases (*lcsQ*), zn-dependent hydrolase (*lcsR*), signal transduction protein (*lcsS*) and nucleoside-diphosphate-sugar epimerase (*lcsT*). The data show that eleven genes are present in *E*. *cladophorae* genome (Figure 2C) with different gene arrangement: *lcsA*, *lcsB*, *lcsE*, *lcsH*, *lcsI*, *lcsK*, *lcsN*, *lcsO*, *lcsP*, *lcsR*, and *lcsT*. Other three genes encoding for tyrosinase, alpha/beta hydrolase and 4-coumarate CoA ligase, along with other five genes encoding for hypothetical proteins are present in this cluster, indicating that this species can be producer of a related compound with a similar biosynthetic mechanism.

Although these BGCs have been found on the genome of *E*. *cladophorae*, none of the resulting compounds were detected in metabolic analysis of the dried crude extracts (Section 3.9). In fact, it has been reported that BGCs may remain silent under laboratory culture conditions and poses a challenge for identifying marine natural products in fungi [57]. To overcome this, different fermentation culture conditions should be used to ‘awake’ or stimulate the expression of silent genes. Different strategies such as the use of epigenetic modifiers, of natural or chemical elicitors and the co-cultivation with other species have been used to induce the production of BGCs coded compounds [57]. This strategy, known as OSMAC (One Strain MAny Compounds) approach, which can activate many silent BGCs in microorganisms to induce the expression of more natural products and of those that are poorly expressed [58].

### 3.7. High-Osmolarity Glycerol (HOG) Pathway

Mitogen-activated protein kinase (MAPK) pathways have been previously identified in *Saccharomyces cerevisiae*, regulating diverse physiological processes such as osmoregulation and nutrient-sensing [59]. These processes are mainly controlled by the high-osmolarity glycerol (HOG) signaling pathway, allowing to adapt to external hyperosmotic stress. In marine fungi, high levels of salinity lead to osmotic and ionic stress [60]. However, it has been proposed that these organisms can maintain positive turgor pressure in a hypertonic environment, through the HOG pathway [61]. This pathway is responsible for regulation of salt efflux pumps and creation of osmolytes compatible with cellular functions [62].

**Figure 3:**
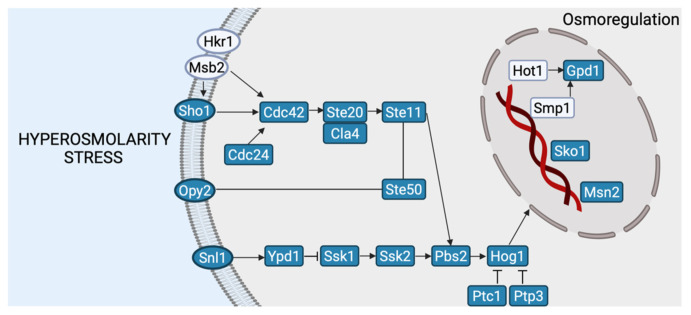
Model illustrating the high-osmolarity glycerol (HOG)—mitogen-activated protein kinase (MAPK) pathway in *Emericellopsis cladophorae* MUM 19.33, based on the *Saccharomyces cerevisiae* HOG-MAPK pathway. Genes that were detected are highlighted in blue. Arrows indicate possible connections. The figure was created with BioRender.com (accessed on 23 September 2021).

The genes essential for the MAPK high osmolarity cascade were identified in the genome of *E*. *cladophorae* (Figure 3). These genes have been previously identified in *S*. *cerevisiae* [59], *Candida albicans* [63], *Aspergillus* spp. [64], *Neurospora crassa* [65], and *Magnaporthe oryzae* [59]. MAPK osmolarity cascade can be activated by osmosensors (*Sho1* and *Sln1*) and response regulator proteins, resulting in *Hog1* activation [66]. *Emericellopsis cladophorae* has the *Sln1* and *Sho1* cascades (*Sln1-Ypd1-Ssk1-Ssk2-Pbs2-Hog1* and *Sho1-Cdc42-Ste20*(or *Cla4*)*-Ste11-Pbs2-Hog1*) to respond to changes in osmolarity in the extracellular environment. The activation of HOG-MAPK pathway ensures the accumulation of a high concentration of glycerol in the cytoplasm to reduce the osmotic pressure and prevent water losses. Also, we identified genes essential for the cascades of MAPK cell wall stress, resulting in cell wall remodeling.

### 3.8. Comparison of Genome Features between E. cladophorae MUM 19.33 and E. atlantica TS7

The genus *Emericellopsis* harbors 23 species described so far. Recently, Hagestad et al. [22] sequenced the first genome of an *Emericellopsis* species: *E*. *atlantica* strain TS7. The genome assembly and gene statistics for *E*. *cladophorae* and *E*. *atlantica* are summarized in Table 4. The *E*. *cladophorae* MUM 19.33 genome size is smaller (1.5%), has a slightly higher GC content (0.3%) and has 14% less genes than *E*. *atlantica* TS7. Both genomes share several conserved genes in biosynthetic gene clusters of ascochlorin, leucinostatin A/B and cephalosporin C. However, some BGCs were species specific, such as those encoding for helvolic acid, botrydial and fusaristatin A in *E*. *atlantica*, while clavaric acid, EQ-4, squalestatin S1 and (-)-Mellein in *E*. *cladophorae*.

The number of predicted genes encoding putative CAZymes of *E*. *cladophorae* is higher (2.7%) than that of *E*. *atlantica*. These differences might define the kind of carbon sources/substrates these species can use, possibly reflecting an adaption to their hosts: as mentioned above *E*. *cladophorae* was isolated from the alga *Cladophora* sp., and *E*. *atlantica* from the sponge *Stelletta normani* [22]. Such carbon sources include typically marine polysaccharides, such as agarose, alginate, carrageenan, chitin, fucoidan, laminarin, ulvan, among others [67]. *Emericellopsis cladophorae* contains enzymes capable to degrade these polysaccharides, such as 4 and 5 genes encoding fucosinases (GH29 and GH95) and ulvanases, respectively. These enzymes are involved in degradation of algal fucoidan and ulvan. Nineteen genes were also detected with fucose activity (GT1 and GT31) and 27 genes encoding chitinases (GH18) and 17 for chitin recognition (CBM18). The presence of specific CAZymes relating to the utilization of marine polysaccharides indicates an adaption to this environment.

### 3.9. Metabolome Analysis

The influence of salt on the metabolomic profile of *E*. *cladophorae* MUM 19.33, was characterized using an untargeted metabolomic approach as described above. Quintuplicate profiles were combined for each condition for comparative analysis. The full list of ions can be found in Table S6. Despite the presence of some unknow compounds, the major classes identified were polyketides, phenolic compounds, terpenes, amino acids, drugs, mycotoxins, carbohydrates, carboxylic acids, fatty acids, alkaloids, and indoles.

**Figure 4:**
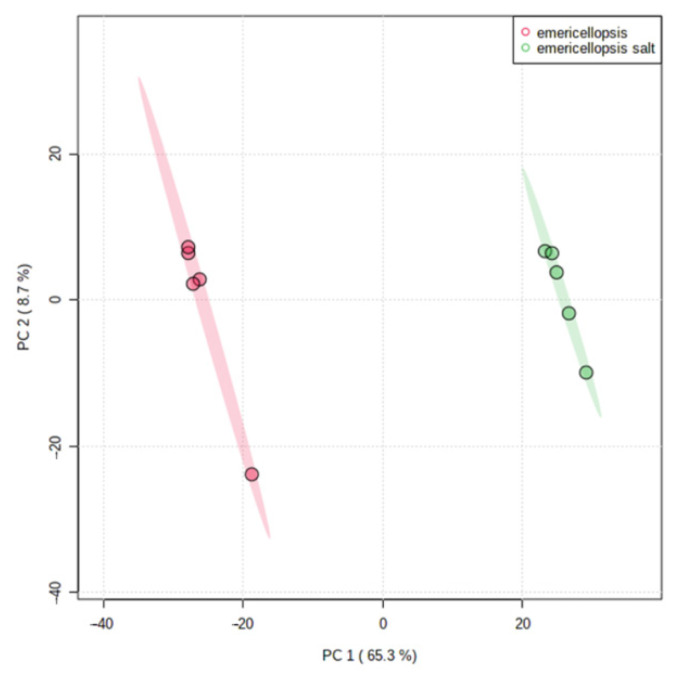
Principal Component Analysis (PCA) scores plot of salted and non-salted extracts of *Emericellopsis cladophorae* MUM 19.33. Green represents salted extracts and in red non-salted extracts.

The scores of the PCA on all filtered compound ions clearly revealed dissimilarities in the metabolome of the salted and non-salted extracts of *E*. *cladophorae* (Figure 4). The growth of *E*. *cladophorae* grows in media with and without sea salt [7] is accompanied by a different metabolic profile, suggesting that this species has the molecular tools to survive in both media.

**Figure 5:**
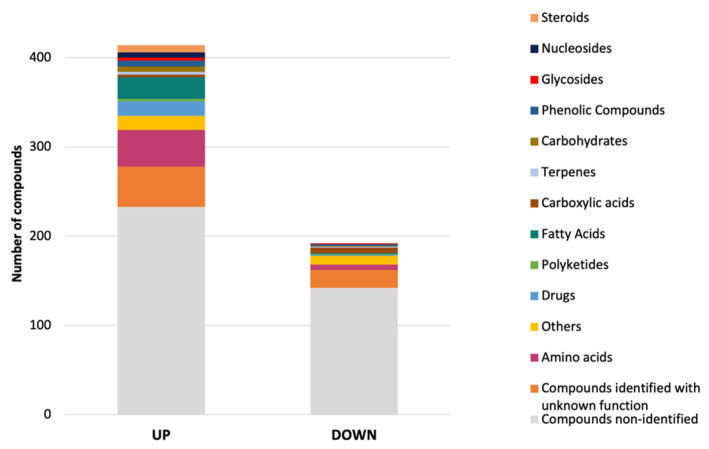
Structural classification of up and down regulated (*p* < 0.01) metabolites produced by *Emericellopsis cladophorae* MUM 19.33, grown in the presence of sea salts.

Subsequently, statistical testing on the filtered compound ions was done using a *t*-test. Computed *p*-values were adjusted using the false discovery rate (FDR) correction. 606 ions having an FDR < 0.01 and a log_2_ fold change (FC) > 2 or <−2 were present in significantly different quantities: 414 and 192 ions were up- and down-regulated in the salted extracts, respectively (Table S7, Figure 5). Due to the lack of information on the MS databases (1 in-house library and MoNA), many of these compounds remain unidentified. We had already suggested [18] that sea salt induces an alteration to the metabolic profile of *E*. *cladophorae*. For example, the compounds ions annotated as ergocryptine, 2’-O-Galloylhyperin, (-)-Gallocatechin 3-gallate, *N*-[1-(4-methoxy-6-oxopyran-2-yl)-2-methylbutyl] acetamide, ferulic acid ethyl ester, 6-Methoxymethylone, 3’-O-Methylguanosine, heptanedioic acid, melezitose, salicylic acid, 5-Sulfosalicylic acid, N4-Acetylsulfadiazine, 5’-Iodoresiniferatoxin, and tafluprost acid were more abundant in salted medium. While the compounds annotated as maltotriose, L-N5-(1-Imino-3-pentenyl)ornithine, hymeglusin, formylciprofloxacin, epiyangambin, lactobionic acid, folinic acid, gatifloxacin, 5-Hydroxymethylcytidine, 4-Hydroxyatorvastatin lactone, and fulvestrant 9-sulfone under non-salted conditions. It is interesting to note that salt induces an increase on the quantity of metabolites produced by *E*. *cladophorae*, specifically of amino acids and peptides. It has been suggested that a selected group of physiologically compliant organic osmolytes, the compatible solutes, amasses when the environmental osmolality is raised in the cytoplasm upon hyperosmotic challenge [68].

Analysis of *E*. *cladophorae* extracts by LC-MS proved effective in detecting bioactive compounds that have been reported for their multiple activities, such as, antibacterial, antifungal, antiviral, anticancer, anti-inflammatory, and antioxidant (Table 5). *Emericellopsis cladophorae* produces diverse metabolites involved in carbohydrate metabolism such as guanosine, maltose, maltotriose, laminaritetraose, palatinose and others, which could be related to the fermentation medium (PDB) that was used. The main constituent of this medium is starch, a complex polysaccharide, widely utilized by fungi to obtain carbon and produce energy. Therefore, when the growth medium contains an excess of carbon source, fungi have the ability to accumulate carbohydrates [69]. Notably, we detected many genes involved in carbohydrate metabolism that were annotated in GO, KEGG and EggNOG databases. For example, in starch and sucrose metabolism, we identified genes encoding for glycosylases, hydrolases and cellulases, glycolysis/gluconeogenesis, pyruvate, amino and nucleotide sugars, galactose, and inositol. In addition, it was also reported in *A*. *niger* a high concentration of citric acid when grown in sugar medium [70], as we also detected in *E*. *cladophorae*.

According to our metabolomic analyses, a large number of amino acids and peptides was detected: Phe, Val-Leu, Pro-Ile, Leu-Gln, Try-Leu and others (Table S5). Considering the GO, KEGG and EggNOG analyses, several genes involved in the amino acid metabolism were found encoding for cysteine and methionine metabolism, glycine, serine and threonine metabolism, alanine, aspartate and glutamate metabolism, phenylalanine, tyrosine and tryptophan biosynthesis, tryptophan metabolism and valine, leucine, and isoleucine biosynthesis.

The number of fungal infections—invasive candidiasis, pneumonia, aspergillosis, cryptococcal meningitis and histoplasmosis—has increased over the last years [71]. With the prevalence of antifungal resistance, it is an urgent and unmet need to develop novel and safe antifungal drugs with novel modes of action, because the few treatments available remain unsatisfactory [72]. We detected some derivative antifungal compounds in our metabolomic analyses such as 9,12,13-Trihydroxyoctadec-10-enoic acid, phosphatidylethanolamine, hymeglusin and salicylic acid. Hymeglusin was also identified in marine-derived fungus *Fusarium solani*, which was isolated from the mangrove sediments, with anti-fungal activities against tea pathogenic fungi *Pestalotiopsis theae* and *Colletotrichum gloeosporioides* [73].

Meir and Osherov [74] suggested that vitamin biosynthesis could be antifungal targets. Several genes from *E*. *cladophorae* were annotated as encoding for metabolism of cofactors and vitamins, particularly of vitamin B1 (thiamine), B2 (riboflavin), B5 (pantothenic acid), B6 (pyridoxine), B7 (biotin) and B9 (folate). Compounds as (-)-Riboflavin and pantothenic acid were also detected in metabolomic analysis. In fungi, these vitamins are important for cellular processes as iron homeostasis [74]. Several filamentous fungi and yeasts, such as *Aspergillus niger*, *A*. *terreus*, *A*. *flavus*, *Penicillium chrysogenum*, and *Fusarium* and *Candida* spp. were reported as natural flavin producers capable of synthesizing riboflavin [75]. A recent study has shown that *Emericellopsis alkalina* produces the antimicrobial peptides Emericellipsins A–E. These compounds have a strong activity against drug-resistant pathogenic fungi (*Aspergillus niger*, *A*. *terreus*, *A*. *fumigatus*, *Candida albicans*, *C*. *glabrata*, *C*. *krusei*, *C*. *tropicalis*, *C*. *parapsilopsis*, *Cryptococcus neoformans* and *Cryp*. *laurentii*), thus indicating its ability act against aspergillosis and cryptococcosis [8]. In a recent study, Gonçalves et al. [18] did not observe antifungal activity against *Candida* spp. in *E*. *cladophorae* extracts, a close relative to *E*. *alkalina* [7]. The observed effects may be related to the fermentation culture conditions or extractions methods. Kuvarina et al. [8] used an alkaline medium (pH 10.5) containing malt and yeast extract with ethyl acetate as solvent to obtain the crude extracts, while Gonçalves et al. [18] used PDB and methanol. Additionally, Hagestad et al. [22] used 11 different fermentation culture media and observed different bioactivities profiles, indicating the likelihood of expression of different compounds.

Antibiotic resistance is a major public health concern and a most serious challenges of our time. In this regard, finding effective solutions to address this problem is crucial. Antibacterial compounds were detected in metabolomic analyses, mainly NovobiocinA and N4-Acetylsulfadiazine. *Emericellopsis cladophorae* genome contains genes involved, not only in the biosynthesis of novobiocin, but also in monobactam, streptomycin, carbapenem, penicillin and cephalosporin biosynthesis. Although these molecules were not detected, we cannot discard the presence of derivative compounds. Apart from biosynthetic gene cluster of Cephalosporin C, the genes mentioned above were also detected in genome of *E*. *atlantica* [22]. However, only penicillins and cephalosporins have been registered as natural products from *Emericellopsis* [76].

The crude extract of *E*. *cladophorae* contains metabolites used in chemotherapy, such as daidzein, daunomycinone, isoreserpin, 3,4-dihydroxycinnamic acid and flavopiridol. To our knowledge, this is the first time that these compounds have been described in a fungus, with exception of 3,4-dihydroxycinnamic acid, which was identified from *Pycnoporus cinnabarinus* [77]. Additionally, rohitukine, a precursor of flavopiridol, was isolated from *F*. *proliferatum*, *F*. *oxysporum* and *F*. *solani* [78]. Hence, the discovery of novel drugs with an increased efficacy for the treatment of different cancers is vital. Therefore, *E*. *cladophorae* could be an interesting candidate to produce compounds used for anticancer therapy.

## 4. Conclusions

This study unveils the genome and the metabolome of the algae-associated fungus, *E. cladophorae* strain MUM 19.33. The genome sequence analysis includes many hypothetical proteins, which is directly related to the lack of sequencing data. Sequencing marine fungal genomes will increase data availability, which can be used as templates for further sequencing analysis. Furthermore, marine fungal genome sequencing allows us to unveil the full biosynthetic potential of compounds with medical, pharmaceutical, and biotechnological applications. Genome sequencing also allows us to disclose fungal biology and specific genomic signatures. To understand the adaptability of fungi to marine environment, an in-depth study is necessary to properly access the function of specific CAZymes related to marine polysaccharides. Our findings also underline that the metabolites produced by *E*. *cladophorae* were responsive to sea salt. Fungal species present in marine environment are expected to have adapted for tolerance to high sodium and chloride concentrations. However, the involvement of these transporters in adaptability mechanisms to osmotic stress in marine fungi is unclear. Also, additional transcriptome and gene expression analyses will help enhance the integrity of this study.

*Emericellopsis cladophorae* is capable of producing a range of antifungal derived compounds, among others. Further studies using are required to confirm the production of these metabolites unraveling the full potential of *E*. *cladophorae*. Also, different culture media, alternative extractions methods and genetic engineering experiments are essential in the future to produce, isolate, and characterize putatively novel compounds.

## Figures and Tables

**Table 1:** General statistics of the *Emericellopsis cladophorae* MUM 19.33 genome assembly, and gene prediction.

	General Features
Genome assembled	26.9 Mb
Number of contigs (>500 bp)	300
Largest contig length	1,489,480 bp
N50	315,653 bp
N75	183,754 bp
GC content	54.34%
Number of predicted genes	8572
Total length of predicted genes	13,253,623 bp
Average length of predicted genes	1546 bp
Total length of predicted genes/Genome assembled	49.2%
Average of exons per gene	3
Average of introns per gene	2

**Table 2:** Statistical results of repetitive sequences and noncoding RNAs for the *Emericellopsis cladophorae* MUM 19.33 genome. SINEs: short interspersed nuclear elements; LINEs: long interspersed nuclear elements; LTRs: long terminal repeats.

Type		Number	Total Length (bp)	Percentage in Genome (%)
Interspersed repeat	SINEs	0	0	0.0000
	LINEs	3	196	0.0007
	LTRs	128	48,958	0.1817
	DNA transposons	22	1362	0.0051
	Rolling-circles	1	37	0.0001
	Unclassified	0	0	0.0000
	Small RNA	62	9505	0.0353
	Satellites	5	698	0.0026
	Simple repeats	4656	183,030	0.6794
	Low complexity	381	17,309	0.0643
	Total	5258	261,095	0.9692
Tandem repeat tRNAs		2365 122	232,036 10,432	0.8614 0.0387

**Table 3:** Genes predicted to code for transporters in the genome of *Emericellopsis cladophorae* MUM 19.33.

Transporter Class	Number of Genes (n)
Channels and pores (TC 1)	461
Electrochemical potential-driven transporters (TC 2)	570
Primary active transporters (TC 3)	405
Group translocators (TC 4)	77
Transmembrane electron carriers (TC 5)	30
Accessory factors involved in transport (TC 8)	270
Incompletely characterized transport systems (TC 9)	384
Total	2197

**Table 4:** Overview of genome assembly and gene statistics for *Emericellopsis cladophorae* MUM 19.33 and *E*. *atlantica* TS7. AA: auxiliary activity; CBM: carbohydrate binding modules; CE: carbohydrate esterases; GH: glycoside hydrolases; GT glycosyltransferases; PL: polysaccharide lyases; NRPS: non-ribosomal peptide synthases; PKs: polyketide synthases.

		*E*. *cladophorae* MUM 19.33	*E*. *atlantica* TS7
Genome assembled		26.9 Mb	27.3 Mb
Coverage		130	225.6
GC content		54.34%	54.2%
Number of genes		8572	9964
Average genes length		1546 bp	1832 bp
Genes encoding CAZymes		407	396
	AA	72	53
	CBM	8	40
	CE	28	21
	GH	200	217
	GT	83	93
	PL	16	17
BGCs		37	35
	NRPS	10	8
	NRPS-like	7	6
	PKs	6	6
	NRPS-PKs	5	3
	NRPS-like-PKs	1	0
	NRPS-PKs-hybrid	0	1
	Terpenes	7	9
	Indole	0	1
	Phosphonate	1	1

**Table 5:** Metabolites with biotechnological potential of *Emericellopsis cladophorae* MUM 19.33 belonging to various chemical classes and related functions. Metabolites were annotated at MSI-level 2. *m*/*z*—ration mass/charge; Rt—retention time (min).

Putative Metabolite	*m*/*z*	Rt	Adduct	Molecular Formula	Class	Function
(-)-Gallocatechin 3-gallate	169.0130	6.87	[M−H-C_15_H_12_O_6_]^−^	C_22_H_18_O_11_	Benzopyrans	Antioxidant activity and inhibitory ability on α-amylase and α-glucosidase related to diabetes mellitus [79]
(-)-Riboflavin	375.1300	8.16	[M−H]^−^	C_17_H_20_N_4_O_6_	Vitamin	Known as vitamin B2 and is the central source of all important flavins [80]. It may be an attractive target for antifungal therapy [81]
2’-O-Galloylhyperin	307.0484	10.47	[M−2H]^−^	C_28_H_24_O_16_	Carboxilic Acid	Antioxidant and anti-inflammatory [82]
3-Isomangostin	427.1782	4.91	[M + OH]^−^	C_24_H_26_O_6_	Xanthone	Derivative of mangostin that has antioxidant, anti-inflammatory, anticancer and anti-microbial activities [83]
3,4-dihydroxycinnamic acid	264.0863	17.01	[M−H]^−^	C_13_H_15_NO_5_	Carboxylic Acid	Antioxidant, anti-cancer, anti-viral and anti- inflammatory [77]
9,12,13-Trihydroxyoctadec-10-enoic acid	329.2324	23.40	[M−H]^−^	C_18_H_34_O_5_	Carboxilic Acid	Antifungal [84]
Citric Acid	191.0185	1.56	[M−H]^−^	C_6_H_8_O_7_	Carboxylic Acid	Antioxidant, preservative, acidulant and pH- regulator [70]
Daidzein	253.0498	14.82	[M−H]^−^	C_15_H_10_O_4_	Flavonoids	Anticancer, anti-inflammatory, protective effects against osteoporosis, diabetes, and cardiovascular diseases [85]
Daunomycinone	379.0825	1.05	[M−H-H_2_O]^−^	C_21_H_18_O_8_	Naphthacene	Antibiotic with anti-cancer activity [86]
(-)-Epigallocatechin	611.1352	2.55	[2M−H]^−^	C_15_H_14_O_7_	Benzopyrans	Antiviral, antimicrobial, antitoxin and anticancer [87]
Ergocryptine	558.0951	16.35	[M−H]^−^	C_32_H_41_N_5_O_5_	Alkaloids	Cause ergot in cereal grains and fescue toxicoses in animals [88]
Flavopiridol	382.0995	3.92	[M−H]^−^	C_21_H_20_ClNO_5_	Piperidines	Treatment of chronic lymphocytic leukemia [78]
Guanosine	282.0838	2.27	[M−H]^−^	C_10_H_13_N_5_O_5_	Nucleosides	Antioxidant, neuroprotective, cardiotonic and immuno-modulatory properties [89]
Hymeglusin	647.3769	21.57	[2M−H]^−^	C_18_H_28_O_5_	Lactones	Fungal beta-lactone antibiotic with anti-fungal activity [73]
Isoreserpin	607.2677	2.33	[M−H]^−^	C_33_H_40_N_2_O_9_	Alkaloids	Anticancer [90]
Laminaritetraose	701.1903	1.10	[M + Cl]^−^	C_24_H_4_2O_21_	Carbohydrates	Obtained from hydrolysis of laminarin, which is a carbohydrate food reserve [91]
N4-Acetylsulfadiazine	291.0537	15.90	[M−H]^−^	C_12_H_12_N_4_O_3_S	Sulfonamide	Marine xenobiotic which is the main constituent of sulfadiazine (antibiotic) [92]
NovobiocinA	611.2305	4.49	[M−H]^−^	C_31_H_36_N_2_O_11_	Glycoside	Antibacterial [93]
Palatinose	341.1078	1.05	[M−H]^−^	C_12_H_22_O_11_	Carbohydrates	Obtained from the enzymatic conversion of sucrose, used in food industries as a sugar substitute [94]
Pantothenic acid	18.1024	3.84	[M−H]^−^	C_9_H_17_NO_5_	Vitamin	Known as vitamin B5 and is essential for fatty acid and carbohydrate metabolism. It may be an attractive target for antifungal therapy [81]
Phosphatidylethanolamine	612.3720	7.33	[M−H]^−^	C_32_H_56_NO_8_P	Glycerophospholipids	Antifungal [95]
Porphobilinogen	225.0870	3.72	[M−H]^−^	C_10_H_14_N_2_O_4_	Pirrole	Involved in the heme biosynthetic pathway and protection from nitrosative stress [96]
Salicylic acid	137.0237	4.12	[M−H]^−^	C7H6O3	Carboxilic Acid	Antifungal [97]

## Data Availability

This Whole-Genome Shotgun project has been deposited in the GenBank database under the accession number JAGIXG000000000. The genome raw sequencing data and the assembly reported in this paper is associated with NCBI BioProject: PRJNA718178 and BioSample: SAMN18524397 within GenBank. The SRA accession number is SRR14127580. Data generated or analyzed during this study are included in this published article and its supplementary information files.

## Supplementary Materials

The following are available online at https://www.mdpi.com/article/10.3390/jof8010031/s1, Table S1: Gene annotation, Table S2: Carbohydrate active enzymes prediction, Table S3: Secreted proteins, Table S4: Transporter’s prediction, Table S5: Biosynthetic Gene Clusters, Table S6: Full list of compounds, Table S7: List of the significantly differential compounds, File S1: matched spectral library compounds.
